# Interleukin-33: A Mediator of Inflammation Targeting Hematopoietic Stem and Progenitor Cells and Their Progenies

**DOI:** 10.3389/fimmu.2013.00104

**Published:** 2013-05-06

**Authors:** Hongnga Le, Wonyoung Kim, Juyang Kim, Hong R. Cho, Byungsuk Kwon

**Affiliations:** ^1^School of Biological Sciences, University of UlsanUlsan, Republic of Korea; ^2^Biomedical Research Center, Ulsan University Hospital, University of UlsanUlsan, Republic of Korea; ^3^Department of Surgery, Ulsan University Hospital, University of UlsanUlsan, Republic of Korea

**Keywords:** IL-33, alarmin, inflammatory cytokine, innate lymphoid cell, hematopoietic stem and progenitor cell, inflammation

## Abstract

Inflammation is defined as a physiological response initiated by a variety of conditions that cause insult to the body, such as infection and tissue injury. Inflammation is triggered by specialized receptors in the innate immune system, which recognize microbial components known as pathogen-associated molecular patterns or endogenous signals produced by damaged cells (damage-associated molecular patterns). IL-33 is a cytokine that is released predominantly at the epithelial barrier when it is exposed to pathogens, allergens, or injury-inducing stimuli. IL-33 target cells are various, ranging from hematopoietic stem and progenitor cells (HSPCs) and essentially all types of their progeny to many non-hematopoietic cells. The pleiotropic actions of IL-33 suggest that IL-33 is involved in every phase of the inflammatory process. In this review, we discuss recent advances in the understanding of how IL-33 orchestrates inflammatory responses by regulating HSPCs and innate immune cells.

## Introduction

Epithelial cells are exposed to external environments on one side, and segregated from the other tissue’s components by the basement membrane on the other side. When they receive extrinsic and intrinsic insults, they release or produce soluble factors that act on stromal cells and immune cells. Damaged cells passively release damage-associated molecular patterns (DAMPs) from the disrupted plasma membrane. DAMPs stimulate adjacent epithelial cells or myeloid cells to secrete inflammatory mediators. Intracellular stores of IL-33 are released from epithelial cells upon infection or injury (Kurowska-Stolarska et al., 2009; Pastorelli et al., 2010; Wills-Karp et al., 2012). This review focuses on how IL-33 regulates inflammation by targeting hematopoietic stem and progenitor cells (HSPCs) and immune cells.

## Expression and Secretion of IL-33

IL-33 was initially found to be highly expressed in the nuclei of high endothelial venules (Baekkevold et al., 2003). Later studies further identified IL-33 as a chromatin-associated nuclear factor inhibiting gene transcription (Carriere et al., 2007; Roussel et al., 2008; Ali et al., 2011). A recent study showed that IL-33 is also found in the cytoplasm (Kakkar et al., 2012). Having been shown to bind to ST2, which is selectively expressed on Th2 cells (Xu et al., 1998; Schmitz et al., 2005), IL-33 was once recognized as a Th2 cytokine. However, its functions far exceed Th2 immunity, as reflected by its broad expression in organs and cell types (reviewed in Mirchandani et al., 2012). IL-33-expressing cells include the stromal and parenchymal cells of organs and immune cells such as macrophages, dendritic cells, mast cells, epithelial cells, smooth muscle cells, fibroblast cells, myofibroblasts, endothelial cells, glial cells, osteoblasts, and adipocytes. Various stimuli are known to induce or upregulate IL-33 expression. In mast cells, IL-33 expression is inducible by the FcεRI signaling pathway that leads to activation of the nuclear factor of activated T cells (NFAT) via Ca^2+^ (Hsu and Bryce, 2012). In dendritic cells, macrophages and fibroblasts, IL-33 mRNA expression can also be induced by TLR stimulation (Talabot-Ayer et al., 2012).

The process by which IL-33 is released from the nucleus is still a matter of debate. Like the other two cytokines in the IL-1 family, IL-1β and IL-18, IL-33 was once thought to be processed by caspase-1, a component of inflammasomes, to become an active form (Schmitz et al., 2005). Yet recent results have demonstrated that full-length IL-33 has full biological activity, and that IL-33 cleavage by caspases results in inactivation of IL-33 (Cayrol and Girard, 2009; Lüthi et al., 2009; Talabot-Ayer et al., 2009; Ali et al., 2010). Although controversy remains as to which type of caspase processes full-length IL-33, it seems that IL-33 is preferentially released from cells during necrosis, and cleaved into inactive forms inside cells during apoptosis (Lüthi et al., 2009). The pattern of IL-33 release is characteristic of endogenous danger signals or alarmins such as the high-mobility group protein B-1 (HMGB-1) and IL-1α (Lamkanifi and Dixit, 2009). The observation that IL-33 is highly expressed in endothelial and epithelial cells which confront environmental threats and are vulnerable to tissue damage also indicates that IL-33 fulfills the requirements of DAMPs, which alert and drive the immune system to manage tissue injury and infection by inducing inflammation (Moussion and Girard, 2008). Indeed, the IL-33 released by endothelial cells of renal microvasculature in response to a necrosis-inducing dose of cisplatin, has a crucial role in exacerbating acute kidney injury (Akcay et al., 2011). However, full-length IL-33 released during inflammation can be proteolytically matured into more bioactive forms by neutrophil elastase and cathepsin G (Lefrancais et al., 2012). Cleaved forms of IL-33 with a greater bioactivity are genuine cytokines that bind to ST2, whilst it has been suggested that full-length IL-33 functions as a DAMP in a ST2-independent way (Luzina et al., 2012). In sum, it seems that IL-33 is passively released from the epithelial barrier which undergo tissue injury, necrosis, and infection and participates in inflammatory processes after taking a more mature form in the extracellular environment with help from neutrophils. It should be noted that chyamase mast cell protease 4, a degranulation product of mast cells, degrades IL-33 in the extracellular matrix and thereby reduce inflammation progression (Waern et al., 2012).

High levels of serum IL-33 are observed in various inflammatory diseases, suggesting that a mechanism exists for the active secretion of IL-33 (Mirchandani et al., 2012). TLRs are typical triggers for the secretion of IL-33. One remarkable *in vivo* example is provided by influenza virus, which promotes IL-33 secretion from alveolar macrophages by stimulating TLR7 signaling (Chang et al., 2011). Indeed, a recent result has shown that mechanical strain in fibroblasts can lead to production of IL-33 without evidence of necrosis which, along with the evidence that IL-33 can traffic between membrane bound-vesicles and the nucleus, suggests that secretion of IL-33 by a classical pathway may be involved (Kakkar et al., 2012). Identifying the signaling pathways leading to IL-33 secretion is an interesting task that has yet to be completed.

## Targets of IL-33

Virtually all types of immune cells and numerous types of non-immune cells respond to IL-33 via its receptor, the ST2-IL-1 receptor accessory protein complex (Mirchandani et al., 2012). The expression pattern of IL-33 receptor indicates that it regulates a complex cellular network consisting of immune cells and non-immune cells.

### Lymphocytes

Lymphocyte subsets capable of responding to IL-33 include Th2 cells, a small population of IL-4-independent atypical Th2 cells (Xu et al., 1998; Kurowska-Stolarska et al., 2008), activated CD8^+^ T cells (Yang et al., 2011; Bonilla et al., 2012), B-1 B cells (Komai-Koma et al., 2011), and NKT cells (Smithgall et al., 2008; Bourgeois et al., 2009; Chen et al., 2012; Kim et al., 2012a). IL-33 stimulates conventional Th2 cells to release IL-4, IL-5, and IL-13 and IL-33-driven Th2 cells profoundly inhibit atherosclerosis (Miller et al., 2008). However, in combination with STAT5-activating cytokines (IL-2, IL-7, and TSLP), IL-33 induces the production of IL-5 and IL-13 but not IL-4 by effector Th2 cells in a TCR-independent fashion (Guo et al., 2009). Even though the significance of this cytokine-induced cytokine production in Th2 cells is unknown (Guo et al., 2012), atypical Th2 and B-1 B cells show the same pattern of cytokine production in response to IL-33 (Kurowska-Stolarska et al., 2008; Komai-Koma et al., 2011). IL-33 polarizes IL-4^−/−^ CD4^+^ T cells into IL-5- and IL-13-producing Th2 cells in the presence of antigen (Kurowska-Stolarska et al., 2008), suggesting that IL-33 may be responsible for the IL-4-independent commitment of Th2 cells. The IL-33-mediated by B-1 cell functions depend largely upon IL-5 that was thought to be produced B-1 cell themselves and other immune cells (Komai-Koma et al., 2011). Similar activity is observed of IL-33 in NKT cells, whose secretion of IFN-γ is induced by IL-33 either alone or in combination with TCR stimulation or IL-12 (Smithgall et al., 2008; Bourgeois et al., 2009). In an airway hypersensitivity model, antigen-stimulated NKT cells induced production of IL-33 by alveolar macrophages, CD11c^+^ dendritic cells and type II pneumocytes, which in turn activated NKT cells to produce IL-13 (Kim et al., 2012a).

### Innate lymphoid cells

Among innate lymphoid cells, NK cells and type 2 innate lymphoid cells (ILC2s) are known to respond to IL-33. NK cells produce IFN-γ as a result of stimulation with IL-33 and IL-12 (Smithgall et al., 2008; Bourgeois et al., 2009). ILC2s are a subset of lymphoid cells only recently identified by many laboratories and referred to by various names. These cells are heterogeneous, but have certain properties in common. They express similar cell surface molecules and secrete IL-5 and IL-13 (Moro et al., 2010; Neill et al., 2010; Price et al., 2010). They also lack hematopoietic lineage markers and express hematopoietic stem cell markers such as Sca-1 and c-Kit, and cytokine receptors such as CD25 (IL-2Rα), CD125 (IL7Rα), IL-17RB (IL-25 receptor), and ST2. All ILC2s respond to epithelial cytokines IL-25, TSLP, and IL-33 and play a pivotal role in anti-helminth responses and airway hypersensitivity in an adaptive immunity-independent fashion.

All types of ILCs are differentiated from Id2-expressing common lymphoid progenitors (CLPs) (Yokota et al., 1999; Moro et al., 2010; Hoyler et al., 2012). The specification of ILC2s is determined by the Th2 transcription factor GATA3 and a retinoic acid-related orphan nuclear receptor family α (RORα) (Halim et al., 2012; Wong et al., 2012). As ILC2s are absent in IL-2 receptor γ chain-deficient mice, IL-2 or IL-7 seems to be required for their maintenance (Moro et al., 2010; Neill et al., 2010). In addition, IL-2 or IL-7 has a synergistic action on both the proliferation of purified ILC2s and their cytokine production with IL-33. However, it is not known yet how *in vivo* administration with IL-25 or IL-33 leads to an enormous increase in ILC2s and the production of high levels of IL-4, IL-5, and IL-13 (Schmitz et al., 2005; Moro et al., 2010; Neill et al., 2010; Price et al., 2010; Saenz et al., 2011).

### Myeloid cells

ST2 is expressed in granulocytes and other myeloid cells which play a role in Th2 immunity. IL-33 treatment induces eosinophilia in mice (Pecaric-Petkovic et al., 2009; Chow et al., 2010; Kim et al., 2010; Stolarski et al., 2010). This is thought to be due to IL-5 production by several types of cells (Kim et al., 2010). The main IL-5 source after IL-33 treatment was recently found to be ILC2-like non-T cells (Ikutani et al., 2012). However, the direct effects of IL-33 on eosinophils are evident. For example, IL-33 induces eosinophil differentiation from bone marrow progenitors through stimulation of IL-5 production in an autocrine fashion (Stolarski et al., 2010). Alone or in cooperation with other cytokines such as GM-CSF, IL-3, or IL-5, IL-33 stimulates the activation of eosinophils to secrete various cytokines and chemokines, upregulates cell adhesion molecules, promotes degranulation, and increases cell survival (Cherry et al., 2008; Suzukawa et al., 2008a; Pecaric-Petkovic et al., 2009; Chow et al., 2010; Stolarski et al., 2010).

ST2 expression is inducible in basophils by IL-3 (Pecaric-Petkovic et al., 2009). Basophils secrete various cytokines and chemokines in response to IL-3 plus IL-33 or an IgE-antigen complex plus IL-33. The types of cytokines and chemokines produced by basophils are similar to those released from ILC2s (i.e., IL-4, IL-5, IL-6, IL-8, IL-9, IL-13, and GM-CSF) (Smithgall et al., 2008; Pecaric-Petkovic et al., 2009; Blom et al., 2011). It seems that the action of IL-33 on basophils is to prime them to respond through main receptors such as the IL-3, IgE, or chemokine receptors (Suzukawa et al., 2008b). The promotion of basophil differentiation by IL-33 depends upon growth factors such as GM-CSF which is produced by bone marrow cells after L-33 stimulation (Schneider et al., 2009). However, some researchers have reported that IL-33 directly upregulates CD11b, IL-4, and IL-6 in basophils (Suzukawa et al., 2008b; Schneider et al., 2009).

IL-33 has significant effects on the biological activities of mast cells which play a major role in Th2 immunity and acute inflammation (reviewed by Lunderius-Andersson et al., 2012). Alone or in combination with SCF, IL-33 promotes mast cell differentiation, maturation, and survival (Allakhverdi et al., 2007; Iikura et al., 2007; Kaieda et al., 2010). There are various mechanisms for the action of IL-33 on mast cells. First, IL-33 may activate mast cells to release various cytokines, chemokines, and lipid mediators without degranulation (Allakhverdi et al., 2007; Ho et al., 2007; Moulin et al., 2007; Silver et al., 2010). Second, IL-33 may enhance the release of these cytokines, chemokines, and lipid mediators from mast cells in a degranulation-dependent or -independent fashion after stimulation with FcεRI or other receptors such as the substance P receptor (Theoharides et al., 2010).

Evidence is accumulating for the various modulatory effects of IL-33 on neutrophils. IL-33 enhances the migratory ability of neutrophils by preventing the downregulation of CXCR2 (Alves-Filho et al., 2010). This occurs because IL-33 inhibits G protein-coupled receptor kinase-1 (GRK-1) downregulation which is induced by TLR signaling during infection by bacteria or fungi (Alves-Filho et al., 2009, 2010; Le et al., 2012). IL-33 also induces the recruitment of neutrophils to sites of infection through the release of chemokines, such as CXCL1, CXCL2, and CCL3, from macrophages (Verri et al., 2010; Le et al., 2012). After exposure to *Candida albicans*, IL-33-primed neutrophils rapidly increase their cell surface complement receptor 3 (CR3: CD11b/CD18) in a TLRs- and Dectin-1-dependent manner, and exhibit higher phagocytic and fungicidal activity (Le et al., 2012). *In vivo*, the biological activity brought about by IL-33 is critical to bacterial and fungal clearance (Alves-Filho et al., 2010; Le et al., 2012).

IL-33 drives macrophages to differentiate into alternatively activated M2 macrophages (Kurowska-Stolarska et al., 2009). IL-13 seems to be required for the IL-33-mediated differentiation of macrophages toward an M2 type, because it induces ST2 expression on the cell surface. Together the two cytokines have a synergistic effect on M2 differentiation (Kurowska-Stolarska et al., 2009). M2 macrophages generated by IL-33 *in vivo* show functional diversity. For example, IL-33-stimulated M2 macrophages play a critical role in the recruitment of leukocytes by producing chemokines (Kurowska-Stolarska et al., 2009), whereas IL-33-polarized M2 macrophages significantly decreased keratitis caused by *Pseudomonas aeruginosa* (Hazlett et al., 2010). The mechanism behind the association of M2 polarization with bacterial or fungal clearance is unknown (Hazlett et al., 2010; Nelson et al., 2011). As IL-33 seems to have little influence on the phagocytic activity of macrophages (Le et al., 2012), its beneficial effects on the host may be twofold: inhibiting hyperresponses to pathogens through M2 polarization, and enhancing the clearance of pathogens by neutrophils. However, responses to IL-33 are likely to vary according to pathogen type (Espinassous et al., 2009).

IL-33 regulates dendritic cells in either a positive or a negative fashion. Cultured bone marrow-derived dendritic cells produce proinflammatory cytokines and upregulate cell surface molecules in response to IL-33. IL-33-primed dendritic cells can efficiently induce allergic airway inflammation (Rank et al., 2009; Besnard et al., 2011). However, rapamycin-conditioned tolerogenic dendritic cells increase ST2 expression in an IL-1β-dependent way and ST2 promotes resistance to the dendritic cell maturation induced by TLR2 or CD40 signaling (Turnquist et al., 2008). In this *in vitro* model, it is not known whether the inhibition of dendritic cell maturation by ST2 depends on IL-33. A recent study has shown that ST2-deficient dendritic cells are overactivated by the presence of antigen, which exacerbates experimental autoimmune encephalomyelitis (Milovanovic et al., 2012).

### Non-hematopoietic cells

IL-33 can function in an autocrine manner in endothelial and epithelial cells, two cell types that produce a large amount of IL-33. IL-33 promotes angiogenesis by increasing endothelial cell proliferation and migration, and induces vascular leakage (Choi et al., 2009). Endothelial cells also secrete inflammatory mediators in response to IL-33 (Yagami et al., 2010), as seen in epithelial cells (Yagami et al., 2010; Fujita et al., 2012). During helminth infection, intestinal epithelial cells induce the production of IL-25, IL-33, and TSLP. This process of induction requires mast cells (Hepworth et al., 2012). IL-25 replaces mast cells in cases of anti-helminth responses. The target cells for IL-25, and presumably the other two cytokines, are the mast cell progenitors that produce IL-5 and IL-9 in response to IL-2 and IL-33 (Hepworth et al., 2012). In ConA-induced hepatitis, NKT cells activate hepatocytes via TRAIL receptors to produce IL-33 (Arshad et al., 2011, 2012). IL-33 in turn has a protective role in hepatitis. Consistent with this, *in vivo* treatment with IL-33 has been shown to decrease liver ischemia-reperfusion injury by preventing the necrosis of hepatocytes with no influence on the production of proinflammatory mediators by Kupffer cells or hepatocytes (Sakai et al., 2012).

### Hematopoietic stem and progenitor cells

**Figure 1:**
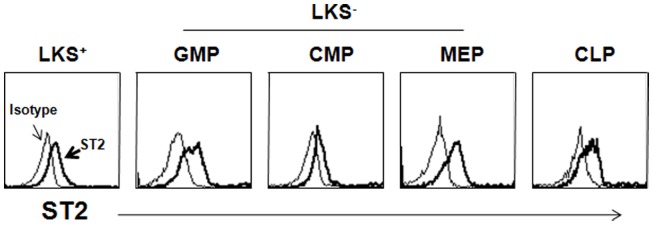
**Expression of ST2 on HSPCs**. Lineage marker-negative cells were enriched from BM suspension before staining with antibodies to hematopoietic subsets and ST2. The open histogram depicts staining with the isotype-matched Ab. LKS^+^, lin^−^c-Kit^+^Sca-1^+^; LKS^−^, lin^−^c-Kit^+^Sca-1^−^; GMP, granulocyte-monocyte progenitor; CMP, common monocyte progenitor; MEP, megakaryocyte-erythrocyte progenitor; CMP, common lymphoid progenitor.

Recent studies have demonstrated that HSPCs participate in inflammation. Although few reports have identified IL-33-mediated biological activity in HSPCs, IL-33 seems to control inflammation by modulating HSPCs either directly or indirectly. ST2 is expressed on hematopoietic stem cells and various subsets of hematopoietic progenitor cells in mice (Figure 1). Helminth infection has shown to accumulate CD34^+^Sca-1^+^ multipotent progenitor cells in the intestine (Hepworth et al., 2012). These cells produce IL-5 and IL-9 by stimulation with IL-2 and IL-33 and differentiate into mast cells in the presence of IL-3 and SCF. Mast cells are required for the production of IL-25, IL-33, and TSLP by intestinal epithelial cells following helminth infection and during subsequent anti-helminth responses (Hepworth et al., 2012). The unknown mast cell-released factors that stimulate epithelial cells may have adjuvant signals that stimulate them to produce sufficiently large quantities of IL-25, IL-33, and TSLP to mount anti-helminth responses. These cytokines orchestrate anti-helminth immunity by activating ILC2s, dendritic cells, Th2 cells, and eosinophils. As IL-25 promotes the accumulation of multipotent progenitor cells in naïve mice, it seems that there is a positive-feedback mechanism to amplify Th2 immunity during helminth infection (Saenz et al., 2011). Similarly, human CD34^+^ hematopoietic progenitor cells produce IL-5, IL-13, GM-CSF, and chemokines in response to IL-33 either alone or in combination with other inflammatory stimuli (Allakhverdi et al., 2009). Because the sputum of asthmatics may contain either IL-5^+^ or IL-13^+^ hematopoietic progenitor cells, these cells are thought to contribute to allergic diseases.

Some HSPCs recirculate constantly between the bone marrow and the periphery for immune surveillance (Massberg et al., 2007). These cells respond to danger signals and differentiate locally into resident innate immune cells in organs (Nagai et al., 2006; Massberg et al., 2007). There is extensive experimental evidence that during the migration and repopulation of HSPCs, chemokine gradients are regulated, along with cell adhesion molecule interactions in the cellular network of hematopoietic niches. In the case of stress situations such as inflammation, massive hematopoiesis occurs in the bone marrow, followed by the large-scale mobilization of HSPCs and both immature and maturing leukocytes into the circulation. The extramedullary hematopoiesis caused by the mobilization of HSPCs is believed to be important for the clearance of pathogens and local inflammation. The administration of IL-33, like that of other inflammatory cytokines (reviewed in Takizawa et al., 2012) induces an excessive accumulation of myeloid cells in the periphery (Schmitz et al., 2005). IL-33 increases myelopoiesis in the bone marrow and the rapid emigration of generated myeloid cells to the periphery, while inhibiting lymphopoiesis by actively expelling CLP cells from the bone marrow (Kim et al., unpublished data). In contrast, IL-33 maintains the number of myeloid progenitor cells in the bone marrow but concomitantly promotes their massive emigration, suggesting that IL-33 plays a role in the generation or proliferation of myeloid progenitor cells in the bone marrow. In the spleens of IL-33-treated mice, IL-33 increases both lymphopoiesis and myelopoiesis (Kim et al., unpublished data).

During IL-23-mediated colitis, hematopoiesis is dysregulated in the bone marrow and spleen (Griseri et al., 2012). IFN-γ induces the accumulation of proliferating HSCs in the bone marrow and spleen. GM-CSF differentiates these cells into granulocyte-monocyte progenitor (GMP) cells at the expense of erythroid and lymphoid progenitors in the spleen. GMPs play a pathogenic role in colitis. Although the producers of IFN-γ and GM-CSF have yet to be identified, IL-23-responding ILC1s and ILC17s are candidates. Because IL-33 also induces a skew toward GMP production, GM-CSF is likely to be involved in this process. Surprisingly, the main producers of GM-CSF may be HSPCs and ILCs (Allakhverdi et al., 2009; Moro et al., 2010; Neill et al., 2010; Monticelli et al., 2011).

**Figure 2:**
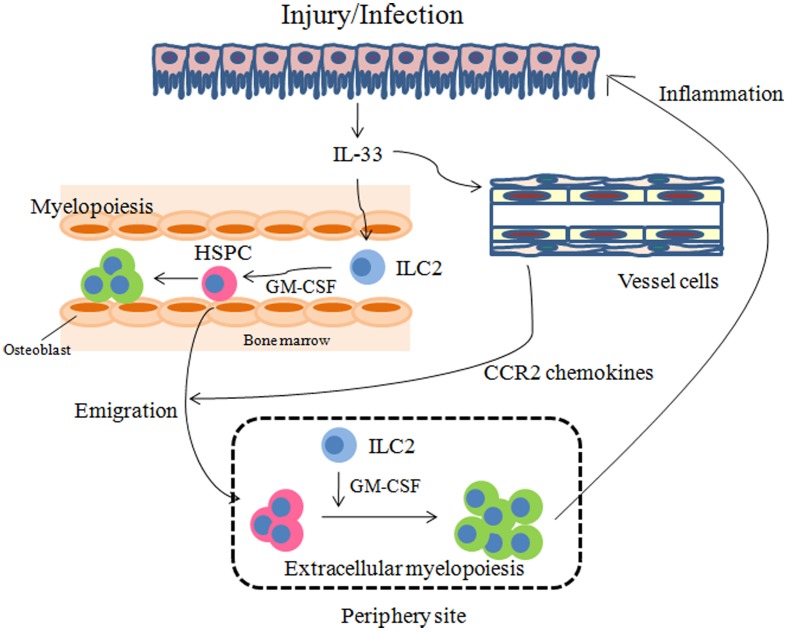
**Models for how IL-33 regulates inflammation through HSPCs**. IL-33 released by injury or infection may stimulate ILC2s to secrete GM-CSF. On the other hand, vessel cells such as endothelial cells and smooth muscle cells may produce CCR2 chemokines, in response to IL-33, which promote the emigration of HSPCs from the bone marrow. GM-CSF in turn may result in myelopoiesis in the bone marrow and periphery to manage injury and infection.

It should be noted that because ILC2s and HSPCs share many cell surface markers and produce similar cytokines such as IL-5, IL-13, and GM-CSF, technical difficulties arise when attempting to distinguish between them. Furthermore, as the administration of IL-25 or IL-33 elicits the expansion of both cell populations *in vivo* in lymphoid organs (Moro et al., 2010; Neill et al., 2010; Saenz et al., 2011), the purification of either subset from IL-25 or IL-33-treated mice cannot exclude the other subset. The only method available to distinguish between these two subsets is to compare their capacity to differentiation into mature hematopoietic cells. At the initial phase of inflammation, ILC2s are likely to respond to IL-33 in organs and as inflammation progresses, HSPCs may be mobilized to inflamed sites and contribute to fulminant inflammation by differentiation into granulocytes and macrophages (models summarized in Figure 2).

## Regulation of Inflammation by IL-33

The IL-33-ST2 system seems to be involved in the regulation of each phase of a generic inflammatory pathway consisting of inducers, sensors, mediators, and effectors (Medzhitov, 2008). Endogenous inducers of inflammation are known to be released from cells or separate tissues after acute tissue injury. As mentioned previously, IL-33 has been proposed to function as an alarmin or DAMP, based on its expression in the epithelium and vascular endothelium whose disruption frequently results in tissue inflammation (Moussion and Girard, 2008; Haraldsen et al., 2009). One report has shown that IL-33 released by necrotic structural cells induces inflammation by activating mast cells to produce proinflammatory cytokines and leukotrienes (Enoksson et al., 2011). Mast cells also play an active role in the production of IL-33 by the helminth-infected intestinal tissue, which promotes their differentiation from Lin^−^CD45^−^ CD34^+^Sca-1^+^ progenitors. This suggests that the mutual regulation of mast cells and intestinal tissue by IL-33 is critical to anti-helminth responses (Hepworth et al., 2012). If IL-33 is secreted from non-necrotic cells upon exposure to various stimuli (Chang et al., 2011; Kakkar et al., 2012), it should be classified as an inflammatory mediator, because its secretion is mediated by inducers of inflammation (Medzhitov, 2008).

There is increasing evidence to suggest that IL-33 is a key inflammatory mediator in a complex network of immune cells and non-immune cells. When tissues receive insults which can lead to inflammation, the major sources of IL-33 may be epithelial cells, endothelial cells, or tissue macrophages (reviewed by Bartemes and Kita, 2012). Research is coming closer to identifying the particular signals that rigger the secretion of IL-33 from the cells. The production of IL-33 by epithelial cells has been reported in many experimental models, including the following: (1) Upon exposure to house dust mites, lung epithelial cells are activated by TLR4 to release IL-1α and IL-1α in turn induces the release of IL-33 in an autocrine fashion (Willart et al., 2012). (2) Similarly, lung epithelial cells secrete IL-33 in response to the fungal allergen *Alternaria alternate* through the induction of ATP release and its subsequent activation of purinergic receptors (Kouzaki et al., 2012). (3) Finally, intestinal epithelial cells release IL-33 in response to helminth infection (Kang et al., 2012). It should be noted that epithelial cells have various mechanism for amplifying inflammation in addition to the initial recognition of danger cells or the production of early endogenous DAMPs (Swamy et al., 2010; Kim et al., 2012b). Disruption of the epithelial cell barrier enables its cells to interact with mesenchymal cells and immune cells, a process known to contribute to the amplification loop of inflammation (Medzhitov, 2008; Kim et al., 2012b). A second source of tissue-cell IL-33 is the macrophage. For example, RNA derived from the influenza virus induces the secretion of IL-33 by alveolar macrophages through TLR7 activation (Chang et al., 2011). Macrophages secrete IL-33 following action by NKT cells and this in turn increases the production of IL-13 by NKT cells and ILC2s (Kim et al., 2012a). Finally, unidentified lung stromal cells were shown to release IL-33 after stimulation of protease-activated receptors with protease allergen papain (Halim et al., 2012).

IL-33 controls inflammation either positively or negatively; in both cases the process is complex. A typical mechanism of IL-33 action on inflammation is to induce the production of additional inflammatory mediators. An IL-33 target cell that has recently received great attention is ILC2 which plays a critical role in Th2 inflammation by secreting IL-5 and IL-13 (Chang et al., 2011; Bartemes et al., 2012; Kim et al., 2012a). IL-5 and IL-13 induce airway eosinophilia and hypersensitivity responses, respectively. As ILC2s express a diverse array of cytokines and chemokines (Moro et al., 2010; Neill et al., 2010; Monticelli et al., 2011), they may be involved in a broad range of inflammatory processes, including healing phases. For example, amphiregulin produced by ILC2s is required for airway remodeling after infection with the influenza virus (Monticelli et al., 2011). Skin fibrosis induced by IL-33 is mediated by IL-13 (Rankin et al., 2010), suggesting that ILC2s may be needed for chronic inflammation. Mast cells are another type of cell involved in IL-33-induced inflammation. In the clinical setting of rheumatoid arthritis, IL-33, secreted by synovial fibroblasts, activates mast cells to produce proinflammatory cytokines (Xu et al., 1998). Mast cells also secrete chemotactic factors for neutrophil migration in response to IL-33 (Enoksson et al., 2012).

IL-33 suppresses inflammation in some clinical settings by driving Th2 immunity. It does this by promoting either M2 macrophage (Kurowska-Stolarska et al., 2009; Hazlett et al., 2010) or Th2 T-cell differentiation (Xu et al., 1998; Kurowska-Stolarska et al., 2008; Groβ et al., 2012). However, the immunosuppressive effects of IL-33 may also be due to its action on regulatory myeloid cells. Dendritic cells are overactivated in the absence of ST2 signaling in experimental autoimmune encephalomyelitis (Milovanovic et al., 2012), suggesting that IL-33 has an immunoregulatory role in these cells. In intravenous immunoglobulin (IVIG)-mediated suppression of inflammation, IL-33 is a key component of the autocrine loop of immunosuppression in regulatory macrophages and dendritic cells (Anthony et al., 2011). The Fc of immunoglobulin induces the production of IL-33 by macrophages through CD209, and IL-33 in turn causes the expansion of IL-4-producing basophils that promote the expression of the inhibitory Fc receptor FcγRIIB on effector macrophages.

Recent studies have revealed IL-33 as a novel survival factor for hepatocytes (Sakai et al., 2012). *In vivo* treatment of IL-33 has been shown to decrease liver ischemia-reperfusion injury by preventing necrosis of hepatocytes with no influence on production of proinflammatory mediators by Kupffer cells or hepatocytes (Sakai et al., 2012).

**Figure 3:**
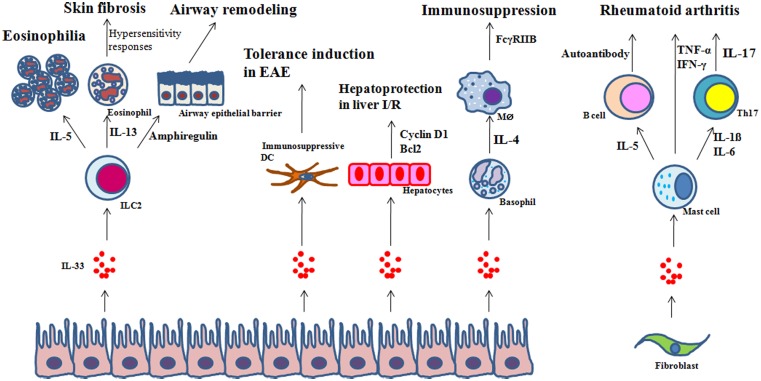
**Examples of regulation of inflammation by IL-33**. In response to IL-33: (1) ILC2s regulate eosinophilia, hypersensitivity and skin fibrosis, or tissue repair through IL-5, IL-13, or amphiregulin, respectively; (2) dendritic cells (DCs) inhibit experimental autoimmune encephalomyelitis (EAE); (3) hepatocytes are protected from ischemia/reperfusion (I/R); (4) basophils secrete IL-4 which in turn promotes the expression of the inhibitory FcγRIIB on effector macrophages; and (5) mast cells produce IL-5, and IL-1β and IL-6 which are involved in autoantibody production by autoreactive B cells and Th17 differentiation, respectively, in rheumatoid arthritis.

It should be noted that IL-33 may be involved in the resolution phase of inflammation. As mentioned above, amphiregulin may be secreted by ILC2 in response to IL-33. In addition, ILC2s express cytokines involved in healing such as IL-13 and TGF-β (Monticelli et al., 2011). Further studies will be needed to define the involvement of IL-33 in the resolution of inflammation. Figure 3 summarizes the roles of IL-33 in inflammation.

## Concluding Remarks

As a broad range of IL-33 targets have been identified, the framework for IL-33 functionality has expanded beyond its well documented role in Th2 immunity. In particular, IL-33 plays a pivotal role in the link between injury at the mucosal barrier and innate immunity. Injuries caused by extrinsic or intrinsic factors result in the release or secretion of IL-33 from the epithelial barrier. ILC2s and HSPCs paly an important role in IL-33-mediated innate responses to injury, parasitic and viral infection, and allergic inflammation. ILC2s and HSPCs produce a variety of mediators involved in inflammatory and defense responses, cell proliferation, and wound healing. Future studies will clarify the elaborate mechanisms by which ILCs and HSPCS control local inflammation.

## Conflict of Interest Statement

The authors declare that the research was conducted in the absence of any commercial or financial relationships that could be construed as a potential conflict of interest.
